# *Coptidis Rhizoma* Suppresses Metastatic Behavior by Inhibiting TGF-β-Mediated Epithelial-Mesenchymal Transition in 5-FU-Resistant HCT116 Cells

**DOI:** 10.3389/fphar.2022.909331

**Published:** 2022-06-13

**Authors:** Yong-Hwi Kang, Jing-Hua Wang, Jin-Seok Lee, Nam-Hun Lee, Chang-Gue Son

**Affiliations:** ^1^ Institute of Bioscience and Integrative Medicine, Daejeon Oriental Hospital of Daejeon University, Daejeon, South Korea; ^2^ Department of Clinical Oncology, Cheonan Oriental Hospital of Daejeon University, Cheonan-si, South Korea

**Keywords:** colorectal cancer, metastasis, 5-fluorouracil, EMT, coptidis rhizoma, TGF-β

## Abstract

Colorectal cancer (CRC) is the second most lethal malignancy worldwide. The high mortality rate of CRC is largely due to cancer metastasis. Recently, suppressing epithelial-to-mesenchymal transition (EMT) has been considered a promising strategy for treating metastatic cancer, especially drug-resistant metastatic cancer. The present study aimed to evaluate the antimetastatic effect of *Coptidis Rhizoma*, as well as the potential underlying mechanisms, using a 5-fluorouracil-resistant colon tumor cell model (HCT116/R). *Coptidis Rhizoma* 30% ethanol extract (CRE) significantly inhibited HCT116/R cells migration and invasion. CRE effectively inhibited EMT in HCT116/R cells by upregulating the expression of an epithelial marker (E-cadherin) and downregulating the expression of mesenchymal markers (vimentin, Snail, and ZEB2) at both the protein and gene levels. Immunofluorescence assays also confirmed consistent patterns in the levels of E-cadherin and vimentin. In addition, the anti-EMT activity of CRE and its related effects were associated with the CRE-mediated suppression of the TGF-β pathway, as shown by changes in the levels of downstream molecules (phosphorylated Akt and p38), and inhibition of migration, invasion, and protein expression of TGF-β after treatment/cotreatment with a TGF-β inhibitor (SB431542). In conclusion, *Coptidis Rhizoma* exerts an antimetastatic effect, especially in the treatment of drug-resistant cancer, and the possible mechanisms are associated with inhibiting EMT *via* TGF-β signaling. Thus, *Coptidis Rhizoma* will likely become a potential therapeutic candidate for simultaneously mitigating drug resistance and metastasis in CRC.

## Introduction

Recently, a global epidemiological investigation revealed that colorectal cancer (CRC) has become the third most common malignancy and it has the second-highest mortality rate (Sung et al., 2021). In 2020, approximately 1.9 million CRC patients were newly diagnosed, and 935,000 CRC-related deaths were reported worldwide (Sung et al., 2021). Due to the lack of clear phenotypic symptoms, early diagnosis and preemptive treatment of CRC patients are difficult (sive Droste et al., 2010). Furthermore, approximately 22% of CRC cases exhibit notably high metastaticity at the time of initial diagnosis, and almost 70% of CRC cases have the potential to metastasize to distant sites during the overall cancer treatment process (Littlejohns et al., 2005; Van Cutsem and Oliveira, 2009; Ansa et al., 2018). In general, metastatic CRC patients have a poor prognosis, with only a 14% 5-years relative survival rate (Wang et al., 2020). Therefore, most clinicians and scholars consider that blocking metastasis is a possible mechanism by which to reduce the cancer mortality rate (Dillekås et al., 2019).

Cancer metastasis is a complex multistep process; in brief, this process includes primary tumor cell invasion and migration, intravasation, movement *via* the circulation, extravasation, and final colonization in other organs (van Zijl et al., 2011; Chei et al., 2019; Dillekås et al., 2019). In this process, epithelial-mesenchymal transition (EMT) plays a critical role in allowing cells to enter systemic circulation, which is the initial step of metastasis (Brabletz et al., 2018). Although several pathways are implicated in the EMT process, transforming growth factor *ß* (TGF-β) signaling is the most well-known regulator of this process (Talbot et al., 2012; Han et al., 2022). Activation of TGF-β leads to EMT in cancer cells *via* the modulation of metastatic phenotype-determining molecules, such as the reduction in E-cadherin expression and the elevation in vimentin expression, which are principal hallmarks of EMT (Attisano and Wrana, 2002; Massagué et al., 2005; Škovierová et al., 2018). A meta-analysis revealed that TGF-β production is positively correlated with distant metastasis and the mortality rate of CRC patients. In particular, both overexpression of TGF-β in tumor tissues and a high level of TGF-β in peripheral blood have been observed in CRC patients characterized by resistance to chemotherapy and distant metastasis (Chen et al., 2017). In general, the EMT and chemoresistance become more obvious during long-term chemotherapy, and these tumors exhibit aggressive metastatic behaviors (Field and Lipton, 2007). The drug resistance-related EMT phenotype includes cells dispersion, pseudopodia development, slow growth, and spindle shape (Yang et al., 2006; Haslehurst et al., 2012; Nagaraju et al., 2014).

Many colon tumors initially respond to fluorouracil- or platinum-based chemotherapy, but most of the tumors develop chemotherapy resistance due to innate or adaptive resistance mechanisms (Tournigand et al., 2004). However, the many aspects of chemotherapy resistance in CRC and the transition to the metastatic phenotype are still unclear (Hu et al., 2019). In the clinic, 5-fluorouracil (5-FU) is a well-known antimetabolite that is frequently used in the treatment of various cancers, including CRC (Longley et al., 2003). Nevertheless, cancer resistance to 5-FU is a critical problem that urgently needs to be solved. In our previous study, 5-FU-resistant HCT116 cells (HCT116/R) modelled the known characteristics of EMT, such as spindle-shaped morphology, loss of intercellular adhesion, and pseudopodia growth (Nagaraju et al., 2014). Accordingly, we reasoned that the HCT116/R cell model could be easily used to evaluate whether any candidates play an antimetastatic roles *via* the modulation of EMT.

On the other hand, *Coptidis Rhizoma* (*C. Rhizoma*) has been traditionally used in Eastern Asia to treat various diseases, such as bacillary dysentery, diabetes, pertussis, sore throat, naphtha, and eczema (Ma et al., 2010; Wang J. et al., 2019). Moreover, previous studies have reported that *C. Rhizoma* displays antitumor activities, such as suppressing cancer cell proliferation, controlling cell cycle arrest, inhibiting tumor growth, and preventing drug resistance *in vitro* and *in vivo* (Wang, 2016; Dan et al., 2017; Mou et al., 2020). We also demonstrated that *C. Rhizoma* reverses 5-FU resistance in HCT116 cells by suppressing thymidylate synthase (TS) (Kang et al., 2021). However, the role of *C. Rhizoma* in metastasis is still unknown.

Hence, we investigated the potential of *C. Rhizoma* to suppress EMT and metastatic behaviors using the HCT116/R cell model. In addition, the novel established 5-FU-resistant cell model needs to be verified whether it could be utilized to evaluate EMT-associated antimetastatic efficacy.

## Materials and Methods

### Preparation of *C. Rhizoma* Extract

The Korean pharmacopeia standard of *C. Rhizoma* was obtained from the Jeong-Seong Traditional Medicine Company (Daejeon, Korea). *C. Rhizoma* powder was mixed with a ten-fold volume of 30% ethanol (1:10; w:v) and continuously shaken for 72 h at room temperature. The supernatants were filtered using Whatman filter paper (Advantec^®^, Tokyo, Japan). Then, the filtrate was concentrated in a rotary evaporator and lyophilized at −70°C, and a final extraction yield of 5.5% (w/w) was acquired. As a qualitative control, fingerprinting analysis of CRE was performed using high-performance liquid chromatography, as described previously (Kang et al., 2021).

### Chemicals and Reagents

RPMI 1640 medium, fetal bovine serum (FBS), Dulbecco’s phosphate-buffered saline (DPBS), penicillin–streptomycin solution, and trypsin-ethylenediaminetetraacetic acid (EDTA) were obtained from WELGENE (Daegu, Korea); TS, E-cadherin, vimentin, Snail, Zinc Finger E-Box Binding Homeobox 2 (ZEB2), TGF-β, Akt, *p*-Akt, and p38 were purchased from Cell Signaling Technology (Danvers, MA, United States); *p*-p38 was obtained from Santa Cruz Biotechnology (Dallas, TX, United States); α-tubulin was obtained from Abcam (Cambridge, MA, United States); SB431542 and berberine were obtained from Sigma–Aldrich (St. Louis, MO, United States); water-soluble tetrazolium salt (WST)-8 cell viability assay kit was obtained from DoGen (Seoul, Korea); bovine serum albumin was obtained from GenDEPOT (Katy, TX, United States); secondary horseradish peroxidase (HRP)-conjugated antibodies were obtained from GeneTex, Inc. (Irvine, CA, United States); and n-butanol was obtained from JT Baker (Mexico City, Mexico).

### High-Performance Liquid Chromatography Analysis

**FIGURE 1:**
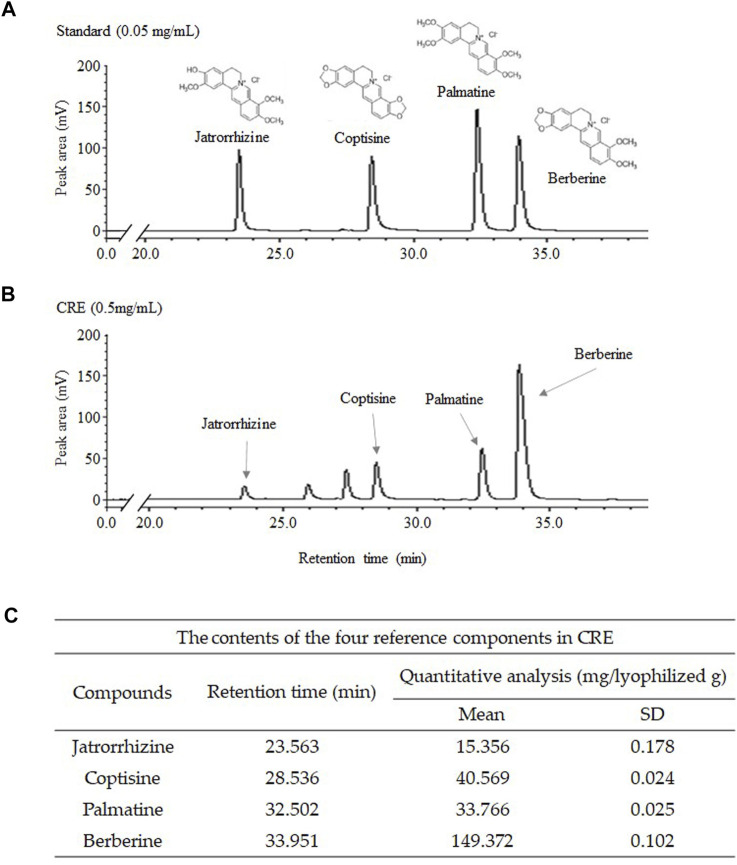
Fingerprint analysis of CRE and standards. CRE and the four reference compounds were analyzed using HPLC–PDA, and the fingerprinting results were acquired at wavelengths of 265 nm **(A,B)**. The level of major compounds in CRE was quantified **(C)**.

Fingerprinting of CRE was performed on an LC-20A Prominence HPLC system (Shimadzu Co., Kyoto, Japan), which consists of binary pumps, an autosampler, and a photodiode array (PDA) detector, at a wavelength of 200–500 nm. CRE was dissolved in 70% methanol and filtered through a 0.2 mm filter (PALL Life Sciences, Ann Arbor, MI, United States). Jatrorrhizine, coptisine, palmatine, and berberine were used as internal reference compounds of CRE for quality control. The C18 column (4.6 × 250 mm, 5 μm, Milford, MA, United States) was used at 30°C. The samples were eluted using 30 mM ammonium bicarbonate and 0.1% (v/v) aqueous triethylamine (A) and acetonitrile (B) with a linear gradient at a 1 ml/min flow rate as follows: 0–15 min, 90%–75% A and 10%–25% B; 15–25 min, 75%–70% A and 25%–30% B; 25–40 min, 70%–55% A and 30%–45% B; 40–45 min, 55% A and 45% B; and 45–60 min, 55%–90% A and 45%–10% B. CRE was detected using a photodiode array at 200–500 nm (Figure 1).

### Cell Culture Conditions and 5-FU-resistant Cell Establishment

HCT116 cells, a human colorectal carcinoma cell line, were cultured in RPMI 1640 medium supplemented with 10% FBS and 100 U/mL penicillin–streptomycin in a 5% CO_2_ atmosphere at 37°C. To establish the 5-FU-resistant cell line (HCT116/R), we followed the protocol described in a previous study. Briefly, parental HCT116 (HCT116/WT) cells were repeatedly treated with 5-FU for 10 months, and the degree of 5-FU resistance in the HCT116/R cells were evaluated by WST-8 assay. According to a previous study, once the survival rate of the HCT116 cells was less than 80%, the concentrations of 5-FU were maintained. Otherwise, the concentration of 5-FU was gradually increased from 2 to 40 µM (Greenwell and Rahman, 2015). HCT116/R cells were maintained in 5-FU-free medium for 1 week prior to use. Finally, RPMI 1640 medium supplemented with 5% FBS was used for all the *in vitro* experiments except the invasion assay.

### Cell Viability Assay

Parental HCT116/WT cells and the derived 5-FU-resistant cells (HCT116/R) were seeded in 96-well microplates at a density of 2 × 10^3^ cells per well. The cells were treated with 5-FU (25 µM) for 48 h. Then, the degree of 5-FU resistance and cytotoxicity of CRE were assessed using a WST-8 assay. HCT116/R cells were treated with four concentrations of CRE (5, 10, 20, and 40 μg/ml) for 48 h, and the results showed that 20 μg/ml is a suitable dose that does not cause significant cytotoxicity. The final absorbance was measured at 450 nm using a spectrophotometer (Molecular Devices, San Jose, CA, United States).

### Western Blotting Analysis

HCT116/R cells were seeded at a density of 3 × 10^5^ cells per dish in 60 mm dishes and were then treated with CRE (20 μg/ml) with/without SB431542 (10 μM, a TGF-β inhibitor) for 48 h. Total protein was extracted by ProPrep™ protein extraction solution (iNtRON Biotechnology, Seongnam, Korea), and the protein concentrations were measured using a bicinchoninic acid (BCA) protein assay kit (Sigma–Aldrich, St. Louis, MO, United States). The isolated proteins were separated by 12% polyacrylamide gel electrophoresis and transferred to polyvinylidene fluoride (PVDF) membranes. After 1 h of blocking with 5% skim milk, the membranes were incubated overnight at 4°C with primary antibodies specific to the following proteins: TS (1:3,000, 9045S, Cell Signaling), E-cadherin (1:1,000, 14472S, and Cell Signaling), vimentin (1:1,000, 5741S, and Cell Signaling), Snail (1:1000S, 3789S, and Cell Signaling), ZEB2 (1:1,000, 97885S, and Cell Signaling), TGF-β (1:1,000, 3711S, and Cell Signaling), Akt (1:1,000, 9272S, and Cell Signaling), *p*-Akt (1:1,000, 9271S, and Cell Signaling), p38 (1:1,000, 9212S, and Cell Signaling), *p*-p38 (1:1,000, SC166182, and Santa Cruz), and α-tubulin (1:1,000, ab7291, and Abcam). After three washes, the membranes were incubated with an HRP-conjugated anti-rabbit (GeneTex and GTX213110-01) or anti-mouse (GeneTex and GTX213110-01) secondary antibody (1:5,000) for 1 h at room temperature. The results were visualized with an advanced enhanced chemiluminescence (ECL) kit, and the band intensities were quantified using ImageJ (Version 1.46, NIH, Bethesda, MD, United States).

### Quantitative Real-Time PCR Analysis

The gene expression of E-cadherin and vimentin was investigated by real-time PCR. HCT116/WT or HCT116/R cells were seeded at a density of 3 × 10^5^ cells per 60 mm dish and were then treated with CRE (20 μg/ml) or vehicle for 24 h. Total mRNA was extracted using TRIzol reagent (Molecular Research Center, Cincinnati, OH, United States), and cDNA was then synthesized using a high-capacity cDNA reverse transcription kit (Ambion, Austin, TX, United States). Then, PCR was performed using SYBR Green PCR Master Mix (Applied Biosystems, Foster City, CA, United States). The primers were as follows: E-cadherin (forward: 5′- CGA GAG CTA CAC GTT CAC GG-3′), reverse: 5′-GGG TGT CGA GGG AAA AAT AGG-3′), vimentin (forward: 5′-TCT GGA TTC ACT CCC TCT GGT-3′), reverse: 5′- CGT GAT GCT GAG AAG TTT CGT-3′), and GAPDH (forward: 5′- CAT GGC CTT CCG TGT TCC T′), reverse: 5′-CCT GCT TCA CCA CCT TCT TGA-3′). Rotor gene Q software from QIAGEN (Hilden, Germany) calculated the relative gene expression. Glyceraldehyde 3-phosphate dehydrogenase (GAPDH) was used as a housekeeping gene.

### Wound Healing Assay

HCT116/WT and HCT116/R cells were seeded in 12-well plates at a density of 2 × 10^5^ cells per well and cultured until they reached confluence. The initial wound was produced by scratching the cell monolayer with a sterile 20-μl pipette tip. Then, the cell debris was removed by washing with DPBS two times. After treatment with CRE (20 μg/ml), SB431542 (10 µM), or berberine (5 μg/ml) for 24 h, image collection was performed using an Olympus IX71 microscope (Olympus, Tokyo, Japan) under a magnification of ×100. The observations were quantified using ImageJ.

### 3D Invasion Assay

HCT116/WT and HCT116/R cells (3 × 10^4^ cells) were embedded in Transwell plates (8 μm pore size, Corning, Acton, MA) coated with 400 μl Matrigel (BD Biosciences). Each type of cell was labeled with 5 μl of DiI (Thermo Fisher Scientific, Waltham, MA). HCT116/WT and HCT116/R cells were plated at identical densities on the gels in 200 μl of RPMI 1640 without FBS. Additionally, RPMI 1640 with 10% FBS (800 μl) was added to the lower chamber of the Transwell. After 12 h of incubation, CRE (20 μg/ml), SB431542 (10 µM), or vehicle was added to the Transwell. After 48 h, the gels were sectioned, and the numbers of invasive cells were analyzed by an Olympus IX71 microscope (Olympus, Tokyo, Japan).

### Immunofluorescence Assay

HCT116/WT and HCT116/R cells were seeded at a density of 3 × 10^5^ cells/well in 12-well plates. After 12 h of incubation, CRE (20 μg/ml) or vehicle was added and incubated for 48 h. After fixation with 4% formaldehyde, the cells were blocked with chicken serum for 15 min. Then, the cells were mixed with E-cadherin (1:200, 14472S, and Cell Signaling), vimentin (1:200, 5741S, and Cell Signaling), and TGF-β (1:200, 3711S, and Cell Signaling) antibodies and incubated overnight. After three washes with DPBS, the cells were incubated with Alexa Fluor 488-conjugated rabbit (1:400, Ab150077, and Abcam) and anti-mouse (1:400, ab150113, and Abcam) antibodies for 1 h at room temperature. The cells were observed using an Olympus IX71 microscope with an Olympus DP74 digital camera (Olympus, Tokyo, Japan).

### Statistical Analysis

In the present study, all the data are expressed as the mean ± standard deviation (SD) and were statistically analyzed by one-way analysis of variance (ANOVA) with post hoc Tukey test using SPSS statistics software (version 20.0 SPSS Inc., Chicago, IL, United States). Differences were considered statistically significant when the *p* value was less than 0.05 or 0.01.

## Results

### Compositional Analysis of Compounds in *C. Rhizoma* Extract

The four major components (jatrorrhizine, coptisine, palmatine, and berberine) of the 30% ethanol extract of *C. Rhizoma* (CRE) were identified by HPLC fingerprinting at a UV wavelength of 265 nm. According to the standard curve, CRE contained 1.536% jatrorrhizine, 4.057% coptisine, 3.377% palmatine, and 14.937% berberine (v/v, Figures 1A–C).

### Characteristics of Epithelial-to-Mesenchymal Transition in 5-FU-Resistant HCT116 Cells

**FIGURE 2:**
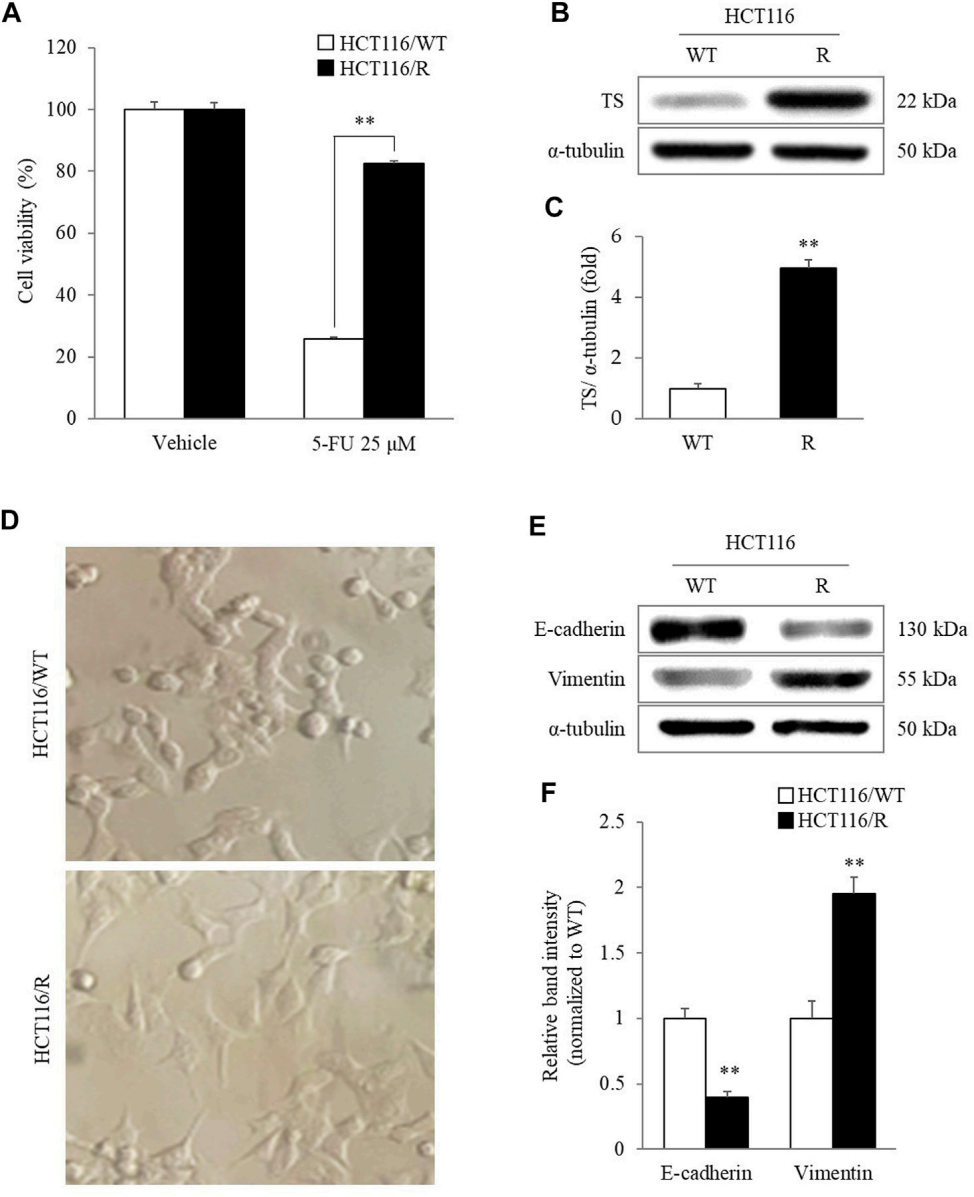
Characteristics of HCT116/R cells. HCT116/R cells showed significant resistance to 5-FU compared to HCT116/WT cells **(A)**. The cellular protein levels of TS were analyzed using western blotting **(B)** and quantified by ImageJ software **(C)**. Characteristics of EMT in HCT116/R cells were observed by assessing morphological changes under a phase-contrast microscope (400 X) **(D)** and by measuring the protein levels of E-cadherin and vimentin using western blotting **(E)** and quantifying these data **(F)**. The data are expressed as the mean ± SD value (*n* = 3). ^**^ *p* < 0.01, compared with HCT116/WT cells. 5-FU, 5-fluorouracil; TS, thymidylate synthase; WT, wild type; R, resistance; EMT, epithelial-mesenchymal transition.

HCT116/R cells showed significant resistance to 25 µM 5-FU-induced cytotoxicity compared to HCT116/WT cells (*p* < 0.01, Figure 1A). In addition, the TS protein level was also markedly higher in HCT116/R cells than in HCT116/WT cells (*p* < 0.01, Figures 1B,C). According to gross morphological observation, HCT116/R cells exhibited obvious phenotypic features that were different from those of HCT116/WT cells, such as a round to spindle cell shape, intercellular separation, and pseudopodia formation (Figure 2D). In addition, the protein level of E-cadherin, an epithelial marker, was significantly decreased in HCT116/R cells compared to HCT116/WT cells. In contrast, the protein level of vimentin showed the opposite trend of the protein level of E-cadherin (*p* < 0.01, Figures 2E,F).

### *C. Rhizoma* Extract Inhibits Metastatic Behaviors in HCT116/R Cells

**FIGURE 3:**
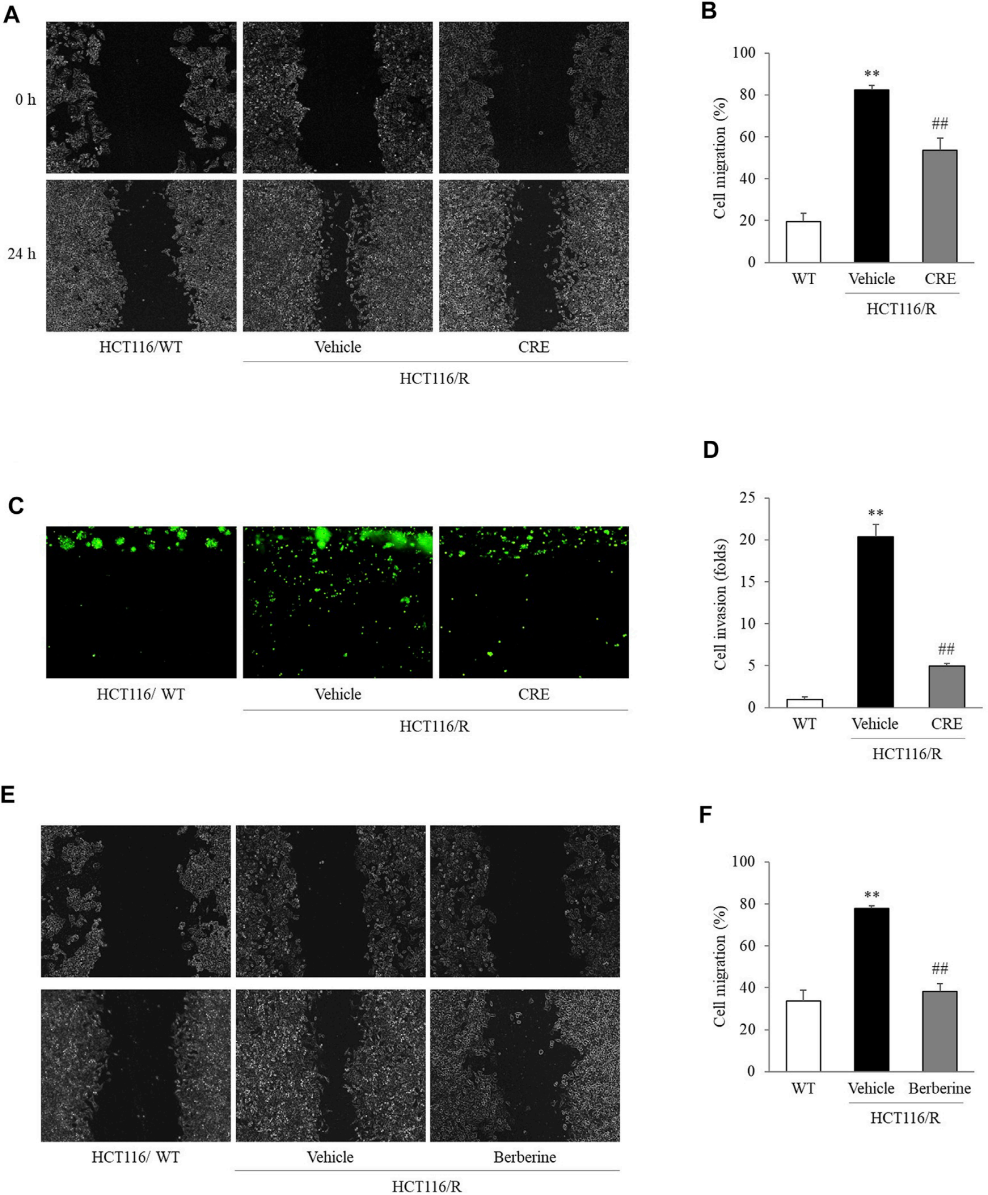
CRE and berberine inhibited the metastatic behaviors of HCT116/R cells. Cell migration was evaluated in a wound-healing assay, and the degree of migration was calculated based on measuring the wound size **(A,B,E,F)**. Cell invasion was assessed by a 3D invasion assay **(C,D)**. All the morphological findings were imaged by an IX71 microscope. The data are expressed as the mean ± SD value (*n* = 3). ^**^ *p* < 0.01, compared to HCT116/WT cells. ^##^ *p* < 0.01 compared to HCT116/R cells.

At the 24 h time point of the wound-healing assay, HCT116/R cells had migrated to fill 82.5% of the initial gap (0 h), and this migration distance was significantly larger than that covered by HCT116/WT cells, which filled 19.6% of the initial gap at 0 h). Then, this accelerated migration behavior of HCT116/R cells was significantly suppressed by CRE (53.4%, 20 μg/ml) compared to non-CRE treatment (*p* < 0.01, Figures 3A,B). Similarly, CRE (20 μg/ml) treatment also distinctly inhibited the invasion of HCT116/R cells compared to the non-CRE treatment in the 3D invasion assay (*p* < 0.01, Figures 3C,D). Meanwhile, 5 µg/ml of berberine also noticeably attenuated the migration behavior in HCT116/R compared to non-CRE treatment (*p* < 0.01, Figures 3E,F).

### *C. Rhizoma* Extract Inhibits Epithelial-to-Mesenchymal Transition Characteristics of HCT116/R Cells

**FIGURE 4:**
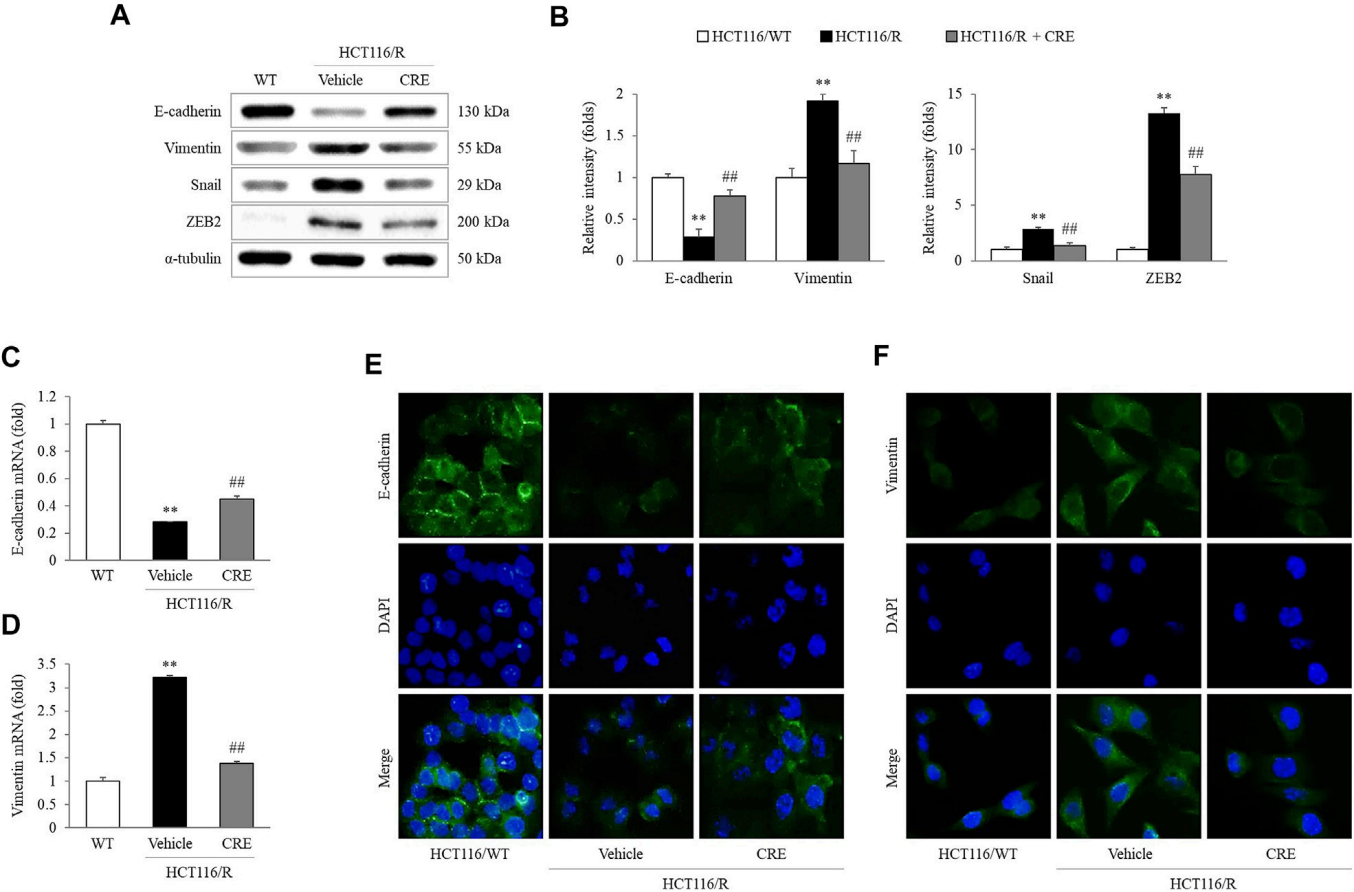
CRE reversed EMT in HCT116/R cells. EMT-related protein levels **(A)** and the corresponding intensities were quantified **(B)**. Gene expression levels of E-cadherin and vimentin were measured in HCT116/WT and HCT116/R cells **(C,D)**. The expression levels of E-cadherin and vimentin were visualized by fluorescence microscopy **(E,F)**. The data are expressed as the mean ± SD value (*n* = 3). ^**^ *p* < 0.01, compared to HCT116/WT cells. ^##^ *p* < 0.01 compared to HCT116/R cells.

Compared to HCT116/WT cells, HCT116/R cells showed lower E-cadherin expression but higher vimentin, Snail, and ZEB2 expression. Moreover, CRE (20 μg/ml) treatment notably upregulated E-cadherin expression and downregulated vimentin, Snail, and ZEB2 expression in HCT116/R cells (*p* < 0.01, Figures 4A,B). Similar effects of CRE (20 μg/ml) on the E-cadherin and vimentin levels were observed when their mRNA expression was measured (*p* < 0.01, Figures 4C,D) and when immunofluorescence was used to assess their protein expression levels (Figures 4E,F).

### *C. Rhizoma* Extract Inhibits Epithelial-to-Mesenchymal Transition *via* the TGF-β Signaling Pathway in 5-FU-Resistant HCT116 Cells

**FIGURE 5:**
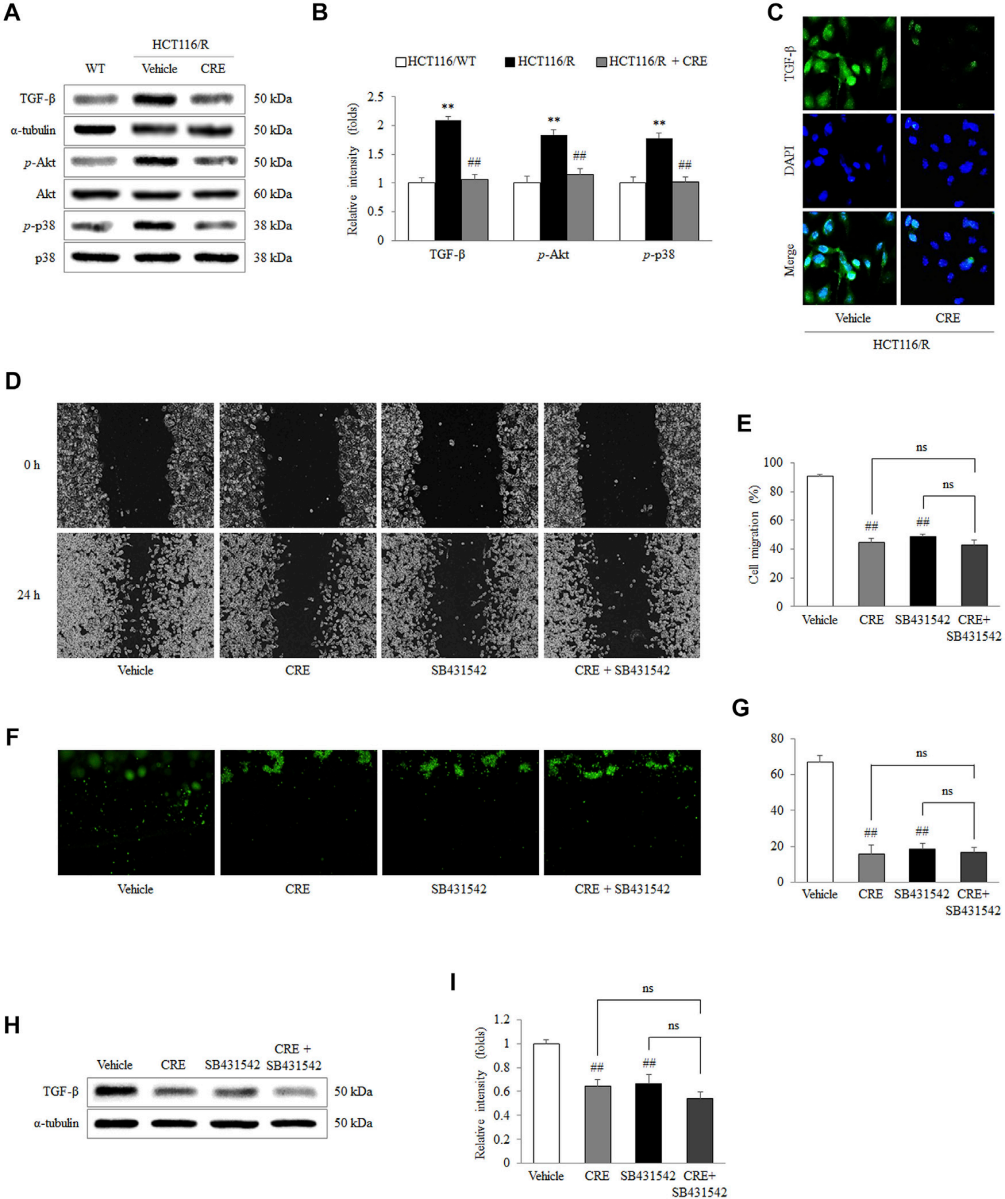
CRE inhibited EMT *via* TGF-β signaling. The expression levels of TGF-β noncanonical pathway-related proteins and the corresponding intensities were quantified **(A,B)**. The level of TGF-β in HCT116/R cells treated with CRE was visualized under fluorescence microscopy **(C)**. The effects of CRE (20 μg/ml) with/without a TGF-β inhibitor (SB431542) on cell migration were analyzed in a wound-healing assay **(D,E)** and 3D invasion assay **(F,G)**. The protein level of TGF-β in HCT116/R cells treated with CRE with/without SB431542 was quantified by western blot **(H)**, and the corresponding intensities were quantified **(I)**. The data are expressed as the mean ± SD value (*n* = 3). ^**^ *p* < 0.01, compared to HCT116/WT cells. ^##^ *p* < 0.01 compared to HCT116/R cells.

In HCT116/R cells, the protein levels of TGF-β and the phosphorylation of Akt and p38 were notably increased compared to those in HCT116/WT cells, whereas CRE treatment (20 μg/ml) significantly reduced the levels of these proteins (*p* < 0.01; Figures 5A,B). In addition, the intensity of the immunofluorescence staining of TGF-β in HCT116/R cells was inhibited by CRE compared to vehicle (Figure 5C). Treatment with both a TGF-β inhibitor (SB431542) and CRE (20 μg/ml) significantly reduced the migration of HCT116/R cells compared to that of vehicle-treated cells (*p* < 0.01, Figures 5D,E). In contrast, their combination (SB431542 plus CRE) did not exert any obviously different effects compared with either drug alone (Figures 5D,E). Furthermore, CRE (20 μg/ml), SB431542, and combination treatment also distinctly inhibited the invasion of HCT116/R cells compared to the none treatment in the 3D invasion assay (*p* < 0.01, Figures 5F,G). Meanwhile, the protein level of TGF-β in HCT116/R cells was also significantly suppressed by CRE, SB431542, and their combination (*p* < 0.01, Figures 5H,I).

## Discussion

Recently, an increasing number of researchers have attempted to identify candidates for cancer treatment in medicinal herbs (Wang et al., 2015). *C. Rhizoma*, a representative antineoplastic herb, has been widely used for a long time to treat various cancers in clinics in east Asia (Yokogawa et al., 2021). Our previous study also demonstrated that CRE could reverse 5-FU resistance in CRC cells by suppressing TS (Kang et al., 2021). To further illustrate the antimetastatic effect of *C. Rhizoma*, an *in vitro* cancer metastasis model was established through long-term treatment of HCT116 cells with 5-FU. 5-FU is a well-known chemotherapeutic drug that inhibits various tumors by blocking DNA replication and cancer cell growth *via* the inhibition of TS, including CRC (Blondy et al., 2020; Zhang et al., 2021). In general, chemotherapy-induced drug resistance in CRC patients is an urgent issue in the clinic (Bathe et al., 1999). Resistance to drugs used to treat cancer also promotes cancer metastasis to the liver, causing high mortality in CRC patients (Popat et al., 2004). In the present study, the viability of HCT116/WT cells was dramatically reduced by 5-FU treatment, whereas HCT116/R cells exhibited significant tolerance to the cytotoxicity caused by 5-FU (Figure 2A). As resistance to 5-FU is principally due to the overexpression of TS (Heerboth et al., 2015), notably high expression of TS in HCT116/R cells compared with HCT116/WT cells confirmed that the cellular model of 5-FU resistance had been successfully established (Figures 2B,C).

Interestingly, HCT116/R cells also underwent morphological alterations after repeated treatment with 5-FU. In detail, HCT116/WT cells exhibited a circular shape with tight cell-cell junctions, whereas HCT116/R cells displayed a morphology characteristic of EMT, such as spindle-like and elongated fibroblastic cell morphologies with loss of intercellular adhesion and increased pseudopodia formation (Figure 2D). EMT is a process that is essential for cancer metastasis and is characterized by the loss of cell-cell adhesion and acquisition of mesenchymal properties by epithelial cancer cells (Heerboth et al., 2015). It is well known that E-cadherin and vimentin are the major indicators of the epithelial and mesenchymal phenotypes, respectively (Heerboth et al., 2015). In our study, significant loss of E-cadherin expression and gain of vimentin expression occurred in HCT116/R cells, but opposite trends were observed in HCT116/WT cells (Figures 2E,F). Consequently, these results indicated that HCT116/R cells acquired EMT features as they developed 5-FU resistance.

In subsequent experiments, 20 μg/ml CRE was administered as an optimal concentration that does not cause cytotoxicity in HCT116/R cells (Supplementary Figure S1A). Although previous studies reported that various compounds from CRE can inhibit migration and invasion in several types of tumors both *in vivo* and *in vitro* (Hambright et al., 2015; Han et al., 2018; Wang et al., 2019b; Quan et al., 2019), the present study first attempted to evaluate the antimetastatic effect of CRE, especially in cells with resistance to anticancer drugs. Increased migration and invasion are two essential behaviors in the early transition stage of cancer metastasis (Kou et al., 2016). As expected, HCT116/R cells exhibited significant enhancement of metastatic behaviors compared to HCT116/WT cells. Notably, CRE treatment noticeably attenuated migration and invasion of HCT116/R cells (Figures 3A–D). These observations demonstrated that CRE has the potential to reverse metastatic behavior in drug-resistant cells. These results might suggest that CRE blocked EMT by increasing E-cadherin and decreasing vimentin, as evidenced by measuring the protein and gene expression levels of these molecules (Figures 4A–D), and confirmed by immunofluorescence results (Figures 4E,F).

We further found that the anti-EMT and antimetastatic effects of CRE involve the modulation of the TGF-β pathway, as shown by the inhibition of TGF-β production by HCT116/R cells (Figures 4A–C). A previous study revealed that 5-FU accelerates EMT *via* activation of TGF-β signaling in CRC cells (Xu et al., 2009). TGF-β induces EMT, supporting metastasis and changes the tumor microenvironment, thereby accelerating cancer progression (Vogelmann et al., 2005; Pickup et al., 2013; Zheng et al., 2022). A clinical meta-analysis reported that a hazard ratio (HR) of 1.68 was calculated between TGF-β expression and overall survival in CRC patients (Chen et al., 2017). Namely, the high expression of TGF-β resulted in a worse prognosis. Moreover, a preliminary clinical study also reported that a human TGF-β monoclonal antibody exerts anticancer effects in patients with advanced malignant melanoma and renal carcinoma (Huynh et al., 2019). Therefore, TGF-β plays a pivotal role in cancer progression, including metastasis. In our study, CRE significantly reduced excessive TGF-β production by HCT116/R cells, as shown by measuring cellular protein levels (Figures 5A,B) and immunofluorescence staining results (Figure 5C). Furthermore, SB431542, a TGF-β inhibitor, exerted a suppressive effect on the migration (Figures 5D,E) and invasion (Figures 5F,G) of HCT116/R cells. The protein level of TGF-β was identically blocked by CRE, SB431542, or their combination (Figures 5H, I). Consequently, we inferred that the prevention of EMT by CRE probably occurs through the inhibition of the TGF-β signaling pathway. In addition, phosphorylation of p38 and Akt facilitates EMT signal transition (Zhang, 2009). Then, ZEB and Snail, which are transcription factors, receive upstream signals to cause the loss of E-cadherin expression (Aigner et al., 2007; Moustakas and Heldin, 2007). CRE notably prevented the decrease in E-cadherin expression through the notable inhibition of downstream TGF-β signaling (*p*-p38 and *p*-Akt) and transcription factor function (Snail and ZEB2) (Figures 4A,B).

Our HPLC fingerprint results (Figure 1A) show that several major compounds in *C. Rhizoma*, such as berberine, jatrorrhizine, coptisine, and palmatine, have been identified quantitatively. As we know, berberine is the major active compound in *C. Rhizoma* (Meng et al., 2018)*.* Identical to the above CRE findings, a previous study revealed that berberine reverses the colon cancer cell induced-EMT process *via* the TGF-β pathway (Huang et al., 2019). When we compared the suppressive effects on the migration capacity in HCT116/R cells, the berberine (5 μg/ml) was more positive than CRE (approximate 3 μg/ml of berberine, Figures 3E,F). Whereas other compounds halted the EMT process/metastasis through distinct mechanisms, for instance, jatrorrhizine suppressed the EMT process through Wnt/β-catenin signaling in a cell, and xenograft mice model (Wang et al., 2019c); coptisine reduced cancer metastasis risk associated with PI3K/AKT signaling pathway *in vivo* and *in vitro* (Li et al., 2014; Huang et al., 2017; Cao et al., 2018); palmatine inhibited the prostate cancer cell invasion through rpS6/NFкB/FLIP (Hambright et al., 2015). Therefore, we postulate that berberine might be an essential contributor to halting the EMT process *via* the TGF-β pathway. Even so, other active compounds against EMT also deserve to be detailedly investigated in the future. Certainly, we do not exclude that the antimetastatic effect of CRE might profit from the synergistic effects of the individual components, at least from berberine in part. The next study will be expected to discover the optimal combination of these ingredients or other function-dominant ingredients. Furthermore, animal and human studies will also be scheduled to confirm the effect and corresponding mechanisms.

## Conclusion

Overall, we conclude that CRE has the potential to inhibit cancer metastasis *via* the suppression of EMT in drug-resistant cells, particularly in CRC. The detailed mechanisms underlying EMT reversion possibly occur through the inhibition of TGF-β overexpression and TGF-β-regulated downstream signaling pathways. Moreover, as a main component of CRE, berberine might be a prime contributor to halting the EMT process among the CRE ingredients. Therefore, we consider that *C. Rhizoma* or berberine will likely become a therapeutic candidate for simultaneously inhibiting drug resistance and metastasis in CRC. In addition, we recommend that the anticancer-drug-resistant cancer cell model is easily applied to screen the antimetastatic candidate or verify the EMT-related mechanisms.

## Data Availability

The original contributions presented in the study are included in the article/Supplementary Materials, further inquiries can be directed to the corresponding authors.
