# Supplementary material for: Coptidis Rhizoma Suppresses Metastatic Behavior by Inhibiting TGF-β-Mediated Epithelial-Mesenchymal Transition in 5-FU-Resistant HCT116 Cells
Source: Front Pharmacol. 2022 Jun 13;13:909331. doi: 10.3389/fphar.2022.909331 (PMC9234293; doi:10.3389/fphar.2022.909331)
Supplement: Supplementary file 1 [file DataSheet1.docx]

Supplementary Material

## Supplementary Figure


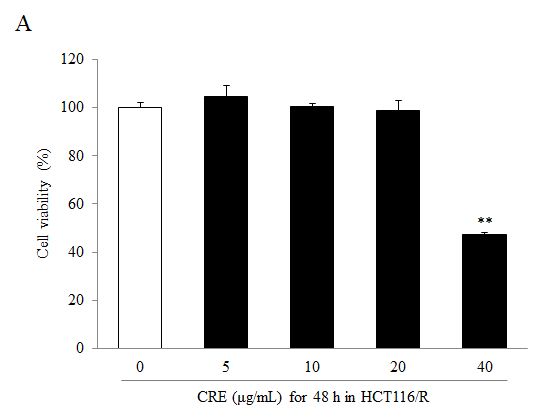


**Supplementary Figure 1.** HCT116/R cells were treated with different concentrations of CRE (A). ^**^*p* < 0.01, compared with non-CRE treated HCT116/R cells.
